# Developmental study of mercury effects on the fruit fly (*Drosophila melanogaster*)

**DOI:** 10.2478/intox-2013-0007

**Published:** 2013-03

**Authors:** Hamideh Abnoos, Masoud Fereidoni, Naser Mahdavi-shahri, Farhang Haddad, Razieh Jalal

**Affiliations:** 1 Department of Biology, Faculty of Sciences, Ferdowsi University of Mashhad, Mashhad, Iran; 2 Department of Chemistry, Faculty of Sciences, Ferdowsi University of Mashhad, Mashhad, Iran

**Keywords:** mercury, fruit fly, larvae, pupae, hatching

## Abstract

Environmental pollution caused by heavy metals such as mercury is one of the most important human problems. It might have severe teratogenic effects on embryonic development. Some pharmacological and physiological aspects of fruit flies (*Drosophila melanogaster*) are similar to humans. So the stages of egg to adult fruit fly, as a developmental model, were employed in the study. Wild adult insects were maintained in glass dishes containing standard medium at 25 °C in complete darkness. Five pairs of 3-day old flies were then transferred to standard culture dishes containing different concentrations of mercury ion. They were removed after 8 hours. We considered the following: The rate of larvae becoming pupae and pupae to adults; the time required for the development; the hatching rate in the second generation without mercury in the culture; the morphometric changes during development in both length and width of the eggs through two generations; larvae, pupae and adult thorax length and width. The results showed that mercury in culture (20–100 mg/l) increase the duration of larvae (*p<*0.01) and pupae (*p<*0.01) development, the rate of larvae becoming pupae (*p<*0.001); pupae maturation (*p<*0.05), the hatching rate (*p<0*.01), the length (*p<*0.05) and width of larvae (*p<*0.01) and pupae (*p<*0.001) and the length in the adult thorax (*p<*0.01) decreased significantly. There was no effect upon the size of eggs. There were also no larvae hatching in concentrations of 200 mg/l of mercury. Negative effects of mercury as a heavy metal are possibly due to the interference of this metal in cellular signaling pathways, such as: Notch signaling and protein synthesis during the period of development. Since it bonds chemically with the sulfur hydride groups of proteins, it causes damage to the cell membrane and decreases the amount of RNA. This is the cause of failure of many enzyme mechanisms.

## Introduction

From the phylogenetic perspective, there is a difference between non-mammalian animals and human beings; however, there are many pharmacologic and physiological similarities with human beings. Among all these animals, the fruit fly (*Drosophila melanogaster*) has the greatest similarity with humans. Thus, it is possible to survey on this basis biochemical, genetic, and physiological causes of many diseases. Accordingly, Drosophila was introduced as a model organism in the twentieth century (Guru Prasad & Hegde, 2010). The Drosophila has a lot of similarities with human beings in some cell signaling pathways, as well as some protein-coding genes. For example, the Notch signaling pathway in the embryonic development of the nervous system in fruit flies and humans are similar (Engel *et al.*, 2012). In addition, we can mention the metallothioneins protein (MTs) in the Drosophila; this protein can bind to heavy metals and reduce their toxicity effects (Al-momani & Massadeh, 2005). These organisms have many advantages in research studies, including fast reproduction, short life cycles, having larval stages, facility in maintenance, and also fewer ethical problems in the work dealing with them (Williams & Beecher, 1944).

In recent decades, attention to the pollution of the environment has been increasing. Heavy metals are one of the most important sources of pollution. Today, the carcinogenicity of arsenic, beryllium, chromium, nickel, cadmium and mercury is known. Pulmonary diseases caused by beryllium and cadmium, as well as lead and mercury-induced neurological disorders are also included. The metals are absorbed in humans primarily through inhalation, food and water (Tchounwou *et al.*, 2012). Among all these heavy metals, mercury is one of the most dangerous environmental pollutant (Anderson, 2003). In addition, mercury is used in the manufacture of many medical devices such as manometers, thermometers, and pace makers (Yaghmaie *et al.*, 2012). It is also used in the pharmaceutical industry to produce many drugs, including disinfectants and diuretics. Mercury is also used in the manufacture of electrical devices such as fluorescent lamps (Hu & Cheng, 2012). In addition, it is used in the chemical industry to make mercury compounds, caustic soda and chlorine (Clarkson & Magos, 2006). Also dentists use mercury in dental filling materials (Bamise *et al.*, 2012). Even inorganic mercury compounds are used in producing pigment, tattoos, dyes, drugs and cosmetics (Chan, 2011), wood preservatives, herbicides, insecticides, pesticides and fungicides. Organic mercury compounds are used as fungicides, seed and pulp (Carpi, 2001). Mercury damages the central nervous system (CNS) regardless of how it enters the body and has irreparable effects on the kidneys (Hazelhoff *et al.*, 2012). Mercury may harm a developing fetus and decrease fertility in men and women (Anderson, 2003). Metallic and inorganic mercury is released into the air and then deposited in the form of methyl mercury. It can infect the lakes that are far from the source of mercury even by hundreds of miles. By entering into the food chain and accumulating in the body of the fish it is later consumed by humans. It must be understood that the mercury concentration accumulated in fish is millions of times greater than its concentration in water. It is thus possible to say that the largest source of this dangerous metal exists in seafood. Mercury is also volatile and rapidly absorbed in the respiratory passages. GI absorption of methyl mercury in humans is about 80% and it is distributed in all tissues, with the greatest concentration in the kidney. Methyl mercury is also capable of crossing the blood-brain barrier and the placenta. Nerve damage in human development has been observed in children whose mothers were exposed to these metals (Alexander, 2008).

Undoubtedly, methyl mercury is very toxic. Experiments in animal, showed that methyl mercury and mercuric chloride (II) reduced spermatogenesis (Sharma & Bhattacharya, 2010). According to the reported toxic effects of mercury and inorganic mercury in industry and the daily life of man, the question is whether inorganic mercury can have an effect on embryonic development or not? To answer this question, the embryonic stages from egg to adult of the fruit fly were chosen as a model to test developmental effects of inorganic mercury on the environment. It was assumed that the mercury concentration in the medium culture of this insect could negatively change the developmental stages, including egg hatching; larvae transformation into pupae, the developmental period and be also the cause of some morphological changes. The techniques used in this study were developed to evaluate the validity of the assumptions on developmental stages of Drosophila.

## Materials and methods

In the present study, the concentrations of zero, 10, 20, 50, 80, 100, 200, 400 mg/l from mercury nitrate (Hg(NO_3_)_2_) in the medium of the fruit fly were used. A medium for this insect includes: white flour (17 g), glucose (15 g), yeast (8.5 g), agar (4 g), ascorbic acid (3 g), propionic acid (3 ml) and distilled water (330 ml).

To prepare it, we first added flour and glucose in a specific dish and then mixed them. After that, we added the yeast and mixed it with the flour and glucose. Subsequently, a volume of 330 ml of distilled water taken from a measuring cylinder was slowly spouted into the mixture until it dissolved well. We waited 30 minutes for the yeast to ferment. Following this stage, we put the exposed dish on a relatively high flame to reach the temperature of 45 °C and then added agar and waited to let it boil. Then we reduced the heat and mixed the medium well until it did not get stuck to the pan. At this stage, it should be left for 25 to 30 minutes exposed to a low flame. In the end, the medium looked like porridge. Subsequently, the pan was removed from the flame to reduce its temperature to 60 °C. Ascorbic acid and propionic acid were added at this stage. A proper fillet must be prepared from gauze and cotton. Then the container was placed in the oven for 60 to 90 minutes to achieve complete sterility. The medium culture was prepared as instructed above; in the meantime, to reduce the temperatures reaching 60 °C, 1 ml of mercury nitrate from the prepared concentrations should be added in sterile containers, and after that, 9 ml of the culture medium was added by a syringe. Using a mixing glass, the material was blended with medium culture.

### Pupation and maturity percentage in different concentrations of mercury nitrate

Five pairs of 3-day-old fruit flies had to be added to the medium culture for mating and egg hatching – and were incubated at 25 °C for eight hours. This time was chosen by the proposed time declared by Ding and Wang (2006). Then, we waited to reach the early age of larvae III (third molt), which were ready to be counted (the method was determined by counting larvae on the screening). After counting the larvae, they should be transferred to second glass beakers (content of the second beaker is exactly similar to the first one). The larvae in the containers were kept in the incubator to become pupae. Pupae of the surface and next to the medium were counted and according to the number of larvae the pupal percentage was calculated. Pupae were maintained in culture dishes inside the incubator, then adults were counted and according to the pupae number, the percentage of maturity was calculated. In addition, it is possible to calculate the percentage of the larvae becoming mature. This was repeated for each concentration seven or eight times.

### Duration of the larval and pupal period

To calculate the length of the larval period, the time started from egg laying to the emergence of the first pre pupae was recorded. The difference between the times shows the length of the larval period. According to the recorded time for the first emerging pupae and also recording the emerging time, duration of the pupal period could be calculated. These tests were repeated for seven or eight times.

### Percentage of hatched eggs in the fruit flies that have grown up in a medium containing mercury

Five pairs of 3-day-old adults, who spent their fetus period in the medium containing mercury and emerged from pupae, were transferred to the medium containing no mercury pollution, and after eight hours, given the opportunity for mating and egg laying, they were exited from the medium. The number of eggs would be counted by Stereomicroscope (egg counting method using a stereomicroscope after a test was selected as the best method). When the larvae were at the beginning of the third molting, they were counted and according to the number of eggs, hatchability percentage was calculated.

### Morphometric changes of eggs and their comparison through two generations

After exiting adults from the medium containing different concentrations of mercury, samples of eggs were photographed and then fixed in small glass dishes containing alcohol 70%.

Of the adults exited after three days, five pairs were cultured in the mercury-free medium. Subsequently, after exiting adults as previously, eggs were sampled and photographed. Then the length and width of the eggs for two generations were measured and the data were also compared.

### Morphometric variation in larvae, pupae and adults

A total of 20 pairs of adult flies (m) were brought upin a flat-bottomed flask of 100 ml. Thus the number of samples was greater and the access to them easier. The first sampling was done with seven larvae (n) 24 hours after emerging of the adults. At this moment, the larvae were at age I (first molting). Afterwards, the sampling was done once every 12 hours. In the controlled sample during the eighth sampling, the size of the larvae would be at the maximum size and developed towards becoming pupae. Therefore, the ninth sampling of pupae would be done. The samples should be photographed as soon as possible, and then the length and width of larvae and pupae was measured and recorded in each sampling. It should be mentioned that it was all carried out according to the recommended method of Day and Wallman (2006). A couple of pupae in media with different concentrations of mercury were allowed to completely emerge from the pupa stage and after three days were anesthetized with ether and kept in 70% alcohol. Dorsal views of adults′ images were taken to measure the length of the chest, with the method described by Agnes and Bundgaard (2000).

### Statistics

The results were presented as mean±SEM. Statistical analysis was performed with GraphPad Prism 5 software. The significant meanings of treatment effects on the groups were done using One-way ANOVA or necessarily Two-way ANOVA and means of data by T_tukey_ tests with a significance level of at least *p*<0.05. Graphs were plotted with Microsoft Excel 2007 software.

## Results

### Results of quantifying developmental studies

Quantitative measurements were carried out to evaluate changes during the length of the developmental period including the length of larval and pupal periods and recording the possible delay caused by any of the developmental stages, effects of heavy-metal ions with the presence of mercury and the success rate of the larvae to pupae, pupae to adults. The results obtained in the experimental group and the control group were compared considering the hatching rate, the insect eggs which spent their embryonic and larval periods in mediums containing mercury versus control group. The probable reduction of the success in each of the above, as the result of heavy metal presence (mercury) was evaluated. The results are shown in Table 1. In concentrations of 200 mg/l and 400 mg/l, no larvae were hatched. Thus quantifying measurements for these concentrations were not done. As shown, the larval and pupal period was increased by mercury ion, demonstrating the delay made in developmental stages of Drosophila. In addition, the pupal percentage (the changing rate of larvae to pupae), the maturity percentage (the changing rare of pupae to adults) and the percentage of eggs hatching were reduced when exposed to mercury.

**Table 1:** Results of quantifying Developmental studies.

Concentration of mercury (mg/l)	Duration of the larval period (hour)	Duration of the pupal period (hour)	Pupation percentage	Maturity percentage	Percentage of hatched eggs
Zero (Control)	108.8±2	88.6±2.2	98.4±0.8	98.9±0.7	79.9±1.6
10	118.9±1.7	89.7±3.7	97.7±1.3	96.3±1.6	73.5±1.8
20	126.4±2.3**	90.3±0.9	90.6±2	86±2.7*	61.6±2.4**
50	150.6±2.3***	94±2.2	83.9±2.7	76.4±2.9***	50±2.9***
80	177.7±2.3***	98.3±1.6	49.9±3.5***	66.3±2.1***	16.4±4.1***
100	193±2.4***	102.6±1.8**	7.7±4***	33.7±3.2***	0

The data were presented as mean±SEM. Compared with the control group, number of replicates, n=7 and the number of pairs of 3-day-old flies, n=5

*** *p<*0.001

** *p<*0.01

* *p<*0.05

### Effect of different concentrations of metallic mercury on larval length and width

**Figure 1:**
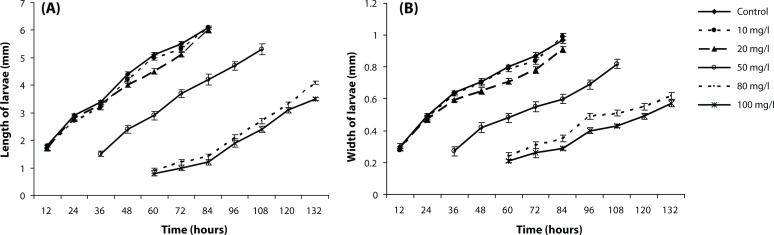
Comparing the mean length (A) and width (B) in larvae at different concentrations of mercury ions during larval growth. In concentration of 50 mg/l, 80 mg/l and 100 mg/l a delay is indicated during the larval sampling in the presence of mercury ions in the medium. Except for the delay, the transverse and longitudinal growth is declining and eventually their growth has not reached its maximum.For the length (F(6,32)=18.203, and *p*<0.001) and the width of larvae (F(6,30)=20.289 and *p*<0.001). The data were presented as mean±SEM (number of larvae n=7 and number of pairs of flies n=20).

Since larval sampling was done every 12 hours, the mean length and width in each measurement is shown as a comparison to the controlled and studied samples (Figure 1). The length and width of larvae exposed to the mercury ion was reduced, for the length (*p*<0.001 and F(6,32)=18.203) and for the width of the larvae (*p<*0.001 and F(6,30)=298.20). In cases where the sampling was not the same, the penetration of mercury concentration in the medium resulted in a delay in molting observed in the larvae at the age of I. Finally, with an increased concentration, the larvae did not grow up to the maximum possible length or width.

### Fruit fly pupae, mean length and width measurements

Following the high concentrations of mercury ions, a significant decrease in mean length and width of the pupae was observed (*p*<0.001).

**Figure 2:**
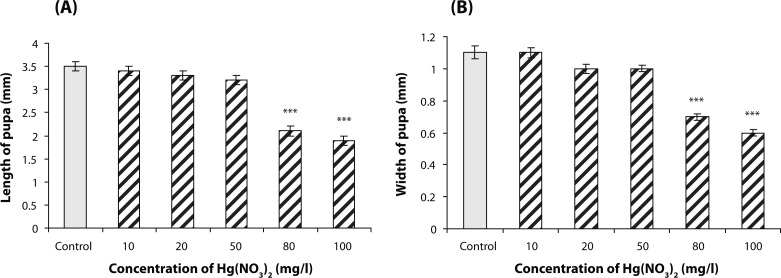
Comparison of the mean of length (A) and width (B) in pupae at different concentrations of mercury. Following the high concentrations of mercury ions (80 and 100 mg/l), a significant decrease in mean length and width of the pupae was observed compared with the control (***p<0.001). The results were presented as mean±SEM (number of pupae, n=7).

The length and width of pupae is affected by high concentrations of mercury, while with concentrations less than 80 mg/l no meaningful effect is seen (Figure 2A and 2B).

### Measuring the chest length in adult fruit flies

**Figure 3:**
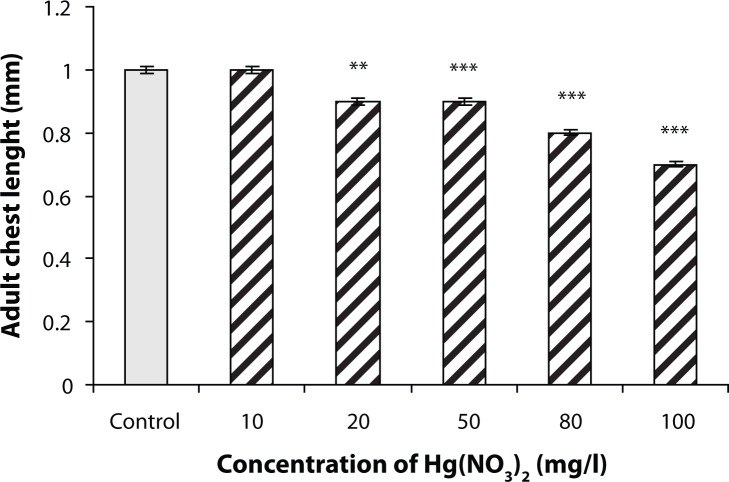
The average size of the adult female at different concentrations of mercury. Following the high concentrations of mercury ions, a significant decrease in mean length of the adult female was observed compared with the control (**p<0.01 and ***p<0.001). The results were presented as mean±SEM (number of adults, n=7).

Following the high concentrations of mercury ions, the average length of the adult body was significantly reduced. The minimum concentration at which the reduction occurred was 20 mg/l (*p*<0.01) (Figure 3).

### Measuring the length and width of eggs

**Figure 4:**
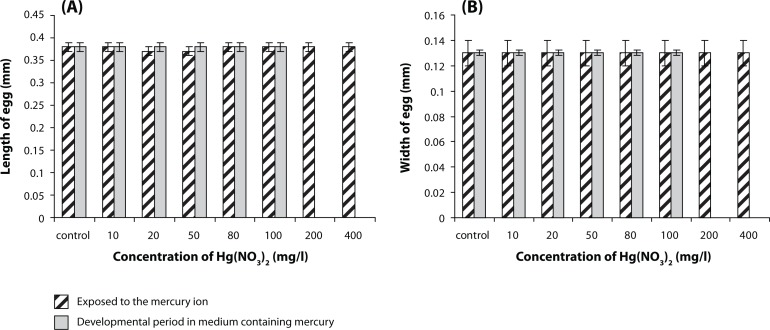
Comparison the length (A) and width (B) of eggs of insects which spent their embryonic and maturation periods in the culture medium containing mercury ions with the eggs of insects where only adults were exposed to the mercury ions. No effect on the length and width of the eggs is evident in both cases. The results were presented as mean±SEM (number of egg n=7).

On comparing the length and width of insects’ eggs which spent their embryonic and maturation periods in the culture medium containing mercury ions with the eggs of insects where only adults had been exposed to the mercury ions it was found that different concentrations of mercury ions had no significant effect on egg size, length and width of the eggs. Despite being in concentrations of 200 and 400 mg/l, only adults that were exposed to mercury ions had fully impaired fetal growth, and no larvae had been hatched (Figure 4A and 4B).

## Discussion

The mechanism of toxic effects of mercury on vertebrates has been identified, yet there was not enough information about the mechanism in invertebrate, until a study by Paula and colleagues was done in 2012 and this matter was discussed.

Paula and colleagues studied mercury effects on mitogen activated protein kinase (MAPK) phosphorylation and anti-oxidant systems in fruit flies. They showed that mercury inhibited functions of the anti–oxidants, acetylcholinesterase (AChE) glutathione s-transferase (GST) and superoxide dismutase (SOD) (Paula *et al.*, 2012). NRF-2 is a gene that can activate many anti oxidants (Osburn and Kensler, 2008). Paula and her colleagues showed that mercury had no effect on the transcription of genes NRF-2, but could greatly reduce the amount of the enzymes SOD and GST. Mercury will probably have an effect on the post-transcriptional steps of these genes.Mercury can also induce phosphorylation of enzymes: extracellular activated protein kinase (ERK) and C-Jun N-terminal kinase (JNK) and also inhibit their effects (Paula *et al.*, 2012). ERK and JNK enzymes are components of the enzyme groups of the MAPK family of serine/threonine protein kinase (Johnson and Lapadat, 2002). Generally, MAPK is responsible for regulation of the mitotic cycle in vertebrates and invertebrates (Sackton *et al*,. 2007). ERK is an enzyme that regulates cell growth and differentiation (Posser *et al.*, 2009). JNK plays a role in the cell cytoskeleton and the formation of Drosophila's cell (Pereira *et al.*, 2011). Thus, inhibition of this enzyme by mercury would disrupt cell growth and differentiation and the formation of cells. Another survey studied extraction of the enzyme AChE from fruit flies and the effect of mercury chlorides. Due to the toxicity of mercury, the enzyme was found to be inhibited (Frasco *et al.*, 2007). Alattia and colleagues conducted a study in 2011; they found that mercury through inhibition of the Notch signaling pathway interfered with γ-secretase (Alattia *et al.*, 2011) in a way which is fundamental in the development of Drosophila's nerve (Engel *et al.*, 2012). This resulted in problems with the development of the Drosophila embryo. In the neural cell culture of Drosophila, increased transcription from two genes, E(SPL)mα and E(SPL)mβ, were affected by methyl mercury, which interfered with the activity of the Notch pathway. However, the activation of these genes is not carried out by inorganic mercury and the Notch pathway can also operate independently (Engel *et al.*, 2012). There is thus the possibility that mercury nitrate is an inorganic mercury involved in the development of the Notch signaling pathway. A stop in the Notch pathway prevents the external growth of axons in the intersegment nerve (ISN). In 2009, it was demonstrated that methyl mercury caused Drosophila to face a problem involved in embryonic development by creating interference in the Notch signaling pathway, and this prevents the larvae from hatching. Neural and glial cell dysfunction was also reported (Rand *et al.*, 2009). In 2009, Baffet and colleagues studied the effects of mercury on Drosophila cell polarity. Germ line cells are surrounded by cells which are called follicular cells. When the follicle cells are exposed to mercury toxicity, they lose their polarity (Baffet *et al.*, 2009). So high concentrations of mercury interfere severely with embryonal development. In 2002, seven heavy metals were found in eggs of birds near the ocean. Mercury was shown to play a significant role in the growth and development of the avian embryo (Burger, 2002). These results are also in line with the present research.

In 1990, Bornias-Vardiabasis and colleagues examined the effects of cadmium and mercury on the embryo of Drosophila. The results suggest that mercury interferes with the differentiation of cells into nerve and muscle. The study concluded that cell differentiation during metamorphosis may also be faced with an interruption or delay (Bornias-Vardiabasis *et al.*, 1990). Mercury, based on its performance, can act as a general poison of cells and protoplasm as it binds chemically with sulfur hydride groups of proteins, causing damage to the cell membrane and decreasing the amount of RNA. This results in failure or delay in the operation of many enzyme systems (Jolley *et al.*, 2000). Flora's research (2008) stated that lead could reduce the number of mitochondrial blades and then ATP synthesis. Since the presence of ATP is essential for the activity of morphometasis of an insect, its deficiency causes a delay in the pupal period. Like lead, mercury operate probably by the same mechanism. This suggestion has yet to be corroborated.

Mercury is likely to have a great effect on the enzymes that are essential for releasing hormones needed for transformation, and it also interferes with their synthesis (Al-Momani and Mossadeh, 2005). Larvae are able to produce resistant proteins against oxidative situations, causing resistance against the toxicity of mercury. The higher the mercury concentration, the less protein is made which reduces the capacity to withstand these stressful conditions (Badre *et al.*, 2005). There are small cysteine-rich proteins (MTs). This protein binds to heavy metals and neutralizes their toxicity of them (Al-Momani and Massadeh, 2005). According to a survey conducted in 2009 by Balamurugon, we found that a transcription factor from metallothionine called MTF1 is activated in response to metals. The agent is able to bind small molecules and to increase DNA transcription (Balamurugon *et al.*, 2009).

As a conclusion, the results indicate that an increased concentration of heavy metal (mercury) is effective in the developmental stages of the fruit fly. In insects that spent their embryonic and maturation period in a medium containing mercury and fed there, their hatching percentage decreased significantly. In relatively high concentrations of mercury ions, developmental processes face severe derangement in the process from larval to adult stages. The interesting thing is that the concentration of 10 mg/l of mercury is highly toxic for animals, including mammals, but does not have a significant influence on the formative stages of fruit flies (National Toxicology Program, 1993). It can be concluded that fruit flies present resistance to this concentration of mercury. Further exploration and understanding of the mechanism of this resistance can suggest new approaches against toxicity with mercury. It is thus not possible to consider fruit flies as bioindicators to evaluate environmental contamination with mercury. Yet they can be a good model to study the effects of toxicity on development.
